# Repositioning: the fast track to new anti-malarial medicines?

**DOI:** 10.1186/1475-2875-13-143

**Published:** 2014-04-14

**Authors:** Julie Lotharius, Francisco Javier Gamo-Benito, Iñigo Angulo-Barturen, Julie Clark, Michele Connelly, Santiago Ferrer-Bazaga, Tanya Parkinson, Pavithra Viswanath, Balachandra Bandodkar, Nikhil Rautela, Sowmya Bharath, Sandra Duffy, Vicky M Avery, Jörg J Möhrle, R Kiplin Guy, Timothy Wells

**Affiliations:** 1 Medicines for Malaria Venture (MMV), PO Box 1826, 20 rte de Pré-Bois, 1215, Geneva 15, Switzerland; 2 Diseases of the Developing World Medicines Development Campus, GlaxoSmithKline, Madrid, Tres Cantos, Spain; 3 Department of Chemical Biology and Therapeutics, St Jude Children’s Research Hospital, Memphis, TN, USA; 4 Independent Consultant, Canterbury, UK; 5 AstraZeneca India Pvt Ltd, Bellary Road, Hebbal, Bangalore, India; 6 Discovery Biology, Eskitis Institute for Drug Discovery, Griffith University, Nathan, Australia

**Keywords:** Malaria, Anti-malarial drugs, Drug repositioning, *in vitro*, *in vivo*, *Plasmodium falciparum*, *Plasmodium berghei*, Candidate drug re-profiling

## Abstract

**Background:**

Repositioning of existing drugs has been suggested as a fast track for developing new anti-malarial agents. The compound libraries of GlaxoSmithKline (GSK), Pfizer and AstraZeneca (AZ) comprising drugs that have undergone clinical studies in other therapeutic areas, but not achieved approval, and a set of US Food and Drug Administration (FDA)-approved drugs and other bio-actives were tested against *Plasmodium falciparum* blood stages.

**Methods:**

Molecules were tested initially against erythrocytic co-cultures of *P. falciparum* to measure proliferation inhibition using one of the following methods: SYBR®I dye DNA staining assay (3D7, K1 or NF54 strains); [^3^H] hypoxanthine radioisotope incorporation assay (3D7 and 3D7A strain); or 4’,6-diamidino-2-phenylindole (DAPI) DNA imaging assay (3D7 and Dd2 strains). After review of the available clinical pharmacokinetic and safety data, selected compounds with low μM activity and a suitable clinical profile were tested *in vivo* either in a *Plasmodium berghei* four-day test or in the *P. falciparum* Pf3D7^0087/N9^ huSCID ‘humanized’ mouse model.

**Results:**

Of the compounds included in the GSK and Pfizer sets, 3.8% (9/238) had relevant *in vitro* anti-malarial activity while 6/100 compounds from the AZ candidate drug library were active. In comparison, around 0.6% (24/3,800) of the FDA-approved drugs and other bio-actives were active. After evaluation of available clinical data, four investigational drugs, active *in vitro* were tested in the *P. falciparum* humanized mouse model: UK-112,214 (PAF-H1 inhibitor), CEP-701 (protein kinase inhibitor), CEP-1347 (protein kinase inhibitor), and PSC-833 (p-glycoprotein inhibitor). Only UK-112,214 showed significant efficacy against *P. falciparum in vivo*, although at high doses (ED_90_ 131.3 mg/kg [95% CI 112.3, 156.7]), and parasitaemia was still present 96 hours after treatment commencement. Of the six actives from the AZ library, two compounds (AZ-1 and AZ-3) were marginally efficacious *in vivo* in a *P. berghei* model.

**Conclusions:**

Repositioning of existing therapeutics in malaria is an attractive proposal. Compounds active *in vitro* at μM concentrations were identified. However, therapeutic concentrations may not be effectively achieved in mice or humans because of poor bio-availability and/or safety concerns. Stringent safety requirements for anti-malarial drugs, given their widespread use in children, make this a challenging area in which to reposition therapy.

## Background

Effective anti-malarial treatment with artemisinin-based combination therapy (ACT) has been critical for supporting and consolidating recent gains in malaria control, with reductions in the number of cases and in mortality [1]. Malaria elimination is becoming a reality for some countries [2], and strategies for global malaria eradication are now being considered [3,4]. This will require new drug regimens with improvements in cost, simplicity and efficacy against resistant strains [5]. In particular, the emergence of *Plasmodium falciparum* strains that are tolerant to artemisinin in the Thai-Cambodia border area is of great concern [6]. This not only has direct implications for artemisinin therapy, but promotes the selection of strains resistant to partner drugs.

New anti-malarial drugs are needed urgently [7]. Recent improvements in cell-based screening technology have led to over 20,000 new starting points in medicinal chemistry [8-10], and the great majority of these data are open access [11]. This has led to a whole series of new molecules in preclinical development [12]. For example, one series, the spiroindolones, has entered early clinical studies only five years after the initiation of screening [13].

In general, however, malaria projects take much longer than five years to go from discovery to having a clinical candidate. Sometimes this is because of technical challenges, but more often because of lack of funding or other resources and the attrition rates are high. It is clearly important to search for new approaches to make this process more efficient. An alternative approach is that of drug repositioning or repurposing. Most simply, this is taking a molecule that has been developed for one indication and showing its utility in another. Although the concept is widely discussed as an attractive drug development strategy, meaningful published data on its success rate and the factors determining that success are limited.

Starting with a molecule that has already undergone clinical trials in another indication provides several potential advantages. The clinical safety profile will be understood, and safe therapeutic doses will have been established. Importantly, human pharmacokinetic data will exist and provide some indication of whether therapeutic concentrations in the new indication can be achieved safely and maintained in patients. In addition, there are regulatory fast track processes, such as the US Food and Drug Administration (FDA) 505 (b) (2) process, where the applicant can rely on data from the studies done by others (with or without the right to reference them) to progress the compound for the new indication. This has acted as a spur to finding new activities of old molecules [14].

Programmes to identify new clinical activities of existing medicines have been conducted in many therapeutic areas, such as oncology [15] and for orphan diseases [16], where there is often an extremely high and specific unmet medical need. Approaches have also been successful in infectious disease, such as tuberculosis [17], schistosomiasis [18] and onchocerciasis [19]. In human African trypanosomiasis, fexinidazole was not so much repositioned as rediscovered following compound mining efforts of more than 700 new and existing nitroheterocycles; efficacy in animal models was initially reported in the 1980s [20,21].

In malaria, there have also been initiatives in drug repositioning. Screening a library of 2,687 compounds containing 1,937 FDA-registered medicines and 750 other molecules in clinical development identified astemizole (a histamine H1 antagonist) as the most promising compound, with good activity against *P. falciparum* blood stages [22]. Unfortunately, this drug was withdrawn because of side effects linked to QTc prolongation, so could not be repositioned as an anti-malarial. A smaller collection of 1,037 existing drugs was tested in an assay for activity against *Plasmodium* liver stages and decoquinate was identified as a potent inhibitor both *in vitro* and *in vivo*[23,24]. As this drug has a veterinary indication, no human safety information is available, but it remains an interesting possibility.

A further potential source of drugs for repositioning is those molecules where clinical development has been discontinued before approval. Of particular interest are drugs that did not achieve efficacy in their proposed indication even though a safe plasma exposure could be obtained in humans. However, it may be difficult to obtain information on such drugs, or gain access to physical samples of them.

In the course of screening large compound collections from pharmaceutical and biotechnology companies against the blood stages of *P. falciparum*[8-10], it was apparent that compounds that had progressed to clinical development were often excluded from the test set. The studies outlined in this paper aimed to specifically identify and test molecules that were not clinically available, but for which some clinical development activity had been conducted. Existing libraries of FDA-approved drugs and some selected bio-actives were also tested, with particular emphasis on antineoplastic and antiretroviral agents. Any compounds showing low micromolar activity and with a suitable pharmacokinetic and safety profile were further evaluated *in vivo*.

## Methods

### Study design

**Figure 1:**
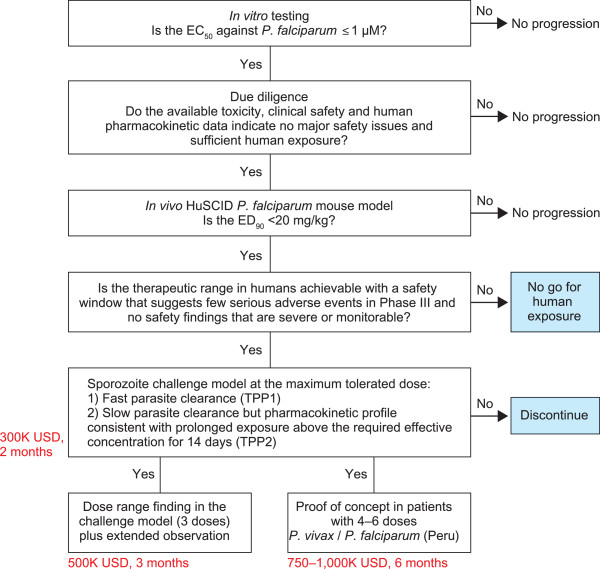
**Medicines for Malaria Venture decision algorithm for repositioning medicines against malaria.** The red text indicates the approximate amounts of money and time that are needed to conduct the studies indicated in the boxes. TPP1: Single exposure radical cure for the treatment of acute uncomplicated malaria in children and adults [25]. TPP2: Non-artemisinin-based combination therapy (NACT) for treatment of acute uncomplicated malaria in children and adults [26].

Figure 1 shows the Medicines for Malaria Venture (MMV) decision algorithm for the repositioning of drugs for the treatment of *P. falciparum* malaria [25,26]. In the studies reported here, compounds were tested *in vitro* against *P. falciparum* and those with significant *in vitro* activity were evaluated based on the data available for toxicity, clinical safety and human pharmacokinetics (due diligence). Compounds that were active *in vitro* and with an acceptable safety/pharmacokinetic profile were progressed to *in vivo* testing. Compound testing sets and assay methods are summarized in Table 1.

**Table 1:** **Summary of compounds tested, *in vitro *** **screening methods, and results**

**Number of compounds**	**Source**	***In vitro *** **testing by:**	***In vitro *** **testing method**	**No. hits (%)**	**No. tested *in vivo*** *
~3,800	• 800 FDA-approved drugs (2008)	SJCRH	• SYBR® I dye DNA staining assay	24 (0.6)	1
	• 2,700 bio-actives (Prestwick, Sigma-Lopac, and MSD)		• *P. falciparum* 3D7 and K1		
	• 296 FDA-approved drugs (2009)				
	• 47 ‘anti-proliferative’ compounds				
63	GSK discontinued clinical candidates	GSK	• Whole-cell [^3^H] hypoxanthine radioisotope incorporation	4 (6.4)	0
			• *P. falciparum* 3D7A		
176	Pfizer STLAR library of discontinued clinical candidates	Discovery biology	• HTS screen using DAPI DNA imaging assay with *P. falciparum* 3D7 and Dd2	5 (2.8)	1
		Pfizer	• EC_50_ determined using SYBR® I dye DNA staining assay with *P. falciparum* 3D7 and K1		
100	AstraZeneca discontinued clinical candidates	AstraZeneca	• SYBR® I dye DNA staining assay	6 (6.0)	2
			• *P. falciparum* NF54		

SJCRH, St Jude’s Children’s Research Hospital; FDA, Food and Drug Administration; GSK, GlaxoSmithKline; HTS, high-throughput screening; DAPI, 4′,6-diamidino-2-phenylindole.

*Two additional compounds were sourced for *in vivo* testing, one each from Novartis and Cephalon Inc. *In vivo* testing was performed only on those compounds that had an acceptable clinical toxicity/safety/pharmacokinetic profile (or no clinical data). Except for AstraZeneca compounds, which were tested in house, all *in vivo* testing was performed by GlaxoSmithKline.

### Compounds screened

An initial set of around 3,500 compounds was assembled and tested by St Jude’s Children’s Research Hospital (SJCRH). This comprised a library of approximately 800 FDA-approved drugs registered up to the year 2008, plus about 2,700 bio-active compounds sourced from the complete Prestwick, Sigma-Lopac and Merck Sharp & Dohme (MSD) libraries. Subsequently, a smaller set of 296 FDA-approved drugs updated for 2009 was tested as well as a small library of 47 ‘antiproliferative’ compounds to further assess targets related to protein kinase inhibitors, antineoplastic and antiretroviral agents. Compounds were not deselected based on known toxicities in order to provide information that could inform the identification and selection of related compounds in development, which could be sourced subsequently. In total, the consolidated test set included approximately 3,800 unique compounds, excluding known anti-malarial drugs. Compounds for the SJCRH screens were sourced firstly from the SJCRH drug repository or, if not available, were obtained from commercial vendors or resynthesized. All supplied compounds were assured by the vendor as >90% pure with quality control data provided and were verified internally at SJCRH after plating.

An initial search of the GlaxoSmithKline (GSK) clinical development pipeline on a commercially available database (Thomson Pharma) revealed around 100 compounds that had been taken into clinical development and subsequently been discontinued. Excluding those molecules that had already been screened against *P. falciparum* in the GSK discovery library [9], samples were obtained from GSK for 63 new compounds. GSK verified samples for purity and activity, and conducted the *in vitro* testing for this compound set.

Pfizer Inc were also approached, and offered to screen their STLAR library of 176 drugs, comprised mainly of pre-Phase III discontinued clinical candidates, though Phase III data were available for a few compounds. There were no approved drugs or active clinical candidates in the set. Pfizer provided samples verified for purity and activity. First, the compound set was tested *in vitro* using high-throughput screening (HTS) by Discovery Biology, Griffith University, Nathan, Australia with subsequent EC_50_ determination by Pfizer in-house.

AstraZeneca (AZ) identified a set of 100 candidate drugs from other therapeutic areas for testing against *P. falciparum*. All 100 candidates had been discontinued for the original indication, and Phase I/II data were available for several compounds. AZ verified the samples for purity and conducted *in vitro* and *in vivo* testing for the compounds.

None of the test sets described above was prescreened for pharmacokinetics/safety but included in their entirety. This was because identification of any active compound could also have led to testing of related follow-up compounds that did not reach clinical testing (and so would not have been included in the initial test set).

### *In vitro* screening assays

More detailed information on the *in vitro* methods is provided in Additional file 1.

SJCRH used the SYBR® I dye DNA staining assay, which measures proliferation of *P. falciparum* in human erythrocytes [27]. *Plasmodium falciparum* strains 3D7 (chloroquine-sensitive) and K1 (chloroquine-resistant) (American Type Culture Collection [ATCC], Manassas, VA, USA) were maintained using established methods [28]. The assay method is as previously described [29]. Tests were run in triplicate in two independent runs to generate ten-point, dose–response curves to determine the half maximal effective concentration (EC_50_) against the 3D7 and K1 *P. falciparum* strains for each drug. EC_50_ values were calculated with the robust investigation of screening experiments (RISE) algorithm with a four-parameter logistic equation. EC_50_ values of <1 μM were considered significant.

GSK Tres Cantos used a whole-cell [^3^H] hypoxanthine radioisotope incorporation assay to determine per cent parasite inhibition at 48 hours and 96 hours [30,31]. *Plasmodium falciparum* 3D7A strain (Malaria Research and Reference Reagent Resource Center MR4; [32]) was maintained as described previously [31]. Parasite growth inhibition assays and EC_50_ determination were carried out following standard methods [31]. Three independent experiments were conducted for each time duration and test compound. Inactive and active controls were also included. Parasite inhibition of ≥50% at 48 hours relative to non-treated parasitized controls was considered significant.

For the Pfizer STLAR set, initial HTS was performed by Discovery Biology, Griffith University, Australia using a 4′,6-diamidino-2-phenylindole (DAPI) DNA imaging assay [33]. *Plasmodium falciparum* 3D7 and the Dd2 clone, which has a high propensity to acquire drug resistance were maintained using standard methods with some adaptations [28,33]. Inhibition values of treated wells were calculated relative to the minimum and maximum inhibition controls [33]. Inhibition of ≥50% at a concentration of 0.784 μM was considered significant. Following the HTS findings, EC_50_ values were determined for a subset of active compounds by Pfizer using a SYBR® I dye DNA staining assay, similar to that described above for SJCRH, using *P. falciparum* 3D7 and K1 (both from David Baker, LSHTM). Per cent anti-malarial activity was calculated relative to the minimum and maximum controls for each of the 11 drug concentrations and EC_50_ values determined from the resulting data plot.

AZ also used a SYBR® I EC_50_ determination assay, but with *P. falciparum* NF54 (MRA-1000, MR4, ATCC, Manassas, VA, USA). The per cent inhibition with respect to the control was plotted against the logarithm of the drug concentration. The curve was fitted by non-linear regression using the sigmoidal dose–response (variable slope) formula to yield the concentration–response curves. The concentration at which 50% inhibition was observed was taken as the EC_50_ value of the compound. A cytotoxicity assay was also performed by AZ, using the human hepatoma Hep G2 cell line and the per cent inhibition and EC_50_ values were calculated as described for *P. falciparum*.

For those compounds showing *in vitro* activity in any of the above tests, the available published and unpublished toxicity, clinical safety and human pharmacokinetic data were reviewed (due diligence).

### *In vivo* assays

Compounds that showed promising activity *in vitro* and that had an acceptable toxicity/safety/pharmacokinetic profile were progressed to *in vivo* testing. For the AZ compound set, a *Plasmodium berghei* four-day suppression test was used. For all other compound sets, activity against *P. falciparum* in the huSCID mouse was determined (as described below). Animal experiments complied with all national and European Union laws, guidelines and codes of conduct for animal care and research use.

#### *Plasmodium berghei* four-day suppression test

AZ compounds were tested by the company for *in vivo* efficacy in a standard four-day suppression test using the rodent malaria parasite *P. berghei*[34]. All animal experimentation protocols were approved by the Institutional Animal Ethics Committee registered with the Government of India (Registration No: 5/1999/CPCSEA). Adult male BALB/c mice (purchased from RCC Laboratories, Hyderabad, India) were used for efficacy studies. Animals were randomly distributed to cages quarantined for one week with veterinary examination and then taken into experimentation. Feed and water were given *ad libitum*. Briefly, male BALB/c mice were infected intraperitoneally with 2×10^7^ infected erythrocytes on day 0. Test compounds were administered orally at a volume of 10 mL/kg as once (UID) or twice daily (BID) doses every 24 hours for four days. On day 3, per cent parasitaemia was estimated microscopically from a Giemsa-stained blood smear. The effect of the test compound on parasite growth was calculated as the difference between the mean value of the control group (taken as 100%) and those of the experimental group and expressed as per cent reduction. Reference anti-malarial compounds (chloroquine and artemisinin) were used as positive controls and the results obtained matched those published in the literature. Pharmacokinetics were analysed in healthy as well as infected mice. Data from healthy mice were used for designing the dosing regimen for the efficacy studies. In infected mice, pharmacokinetics was carried out on day 2 of compound administration. One mouse per time point was sampled according to the fast mouse pharmacokinetic protocol [35].

#### *Plasmodium falciparum* huSCID mouse model

*In vivo* testing using this model was performed by GSK at Tres Cantos, against *P. falciparum* 3D7 (*in vivo* strain Pf3D7^0087/N9^generated by GSK using Pf3D7 obtained from Eduardo Dei-Cas, Institute Pasteur, Lille, France [36]) growing in peripheral blood of female NOD-*scid IL-2Rγ*^null^ mice engrafted with human erythrocytes, i e, a ‘humanized’ mouse model, following published protocols [36,37]. Briefly, animals were infected intravenously with 20×10^6^ infected erythrocytes on day 0. Test compounds were administered orally at a volume of 20 mL/kg or subcutaneously (10 mL/kg) in an appropriate inactive vehicle. Dosing was initiated at the maximum tolerated dose in mice on day 3 after infection and continued once daily for four days. Each experimental group was n = 3 mice unless otherwise stated. Control animals received vehicle only and a quality control assay used chloroquine at target doses of 3 mg/kg and 7 mg/kg. Venous blood samples for parasitology (2 μL) were taken at days 3, 5, and 7 after infection. Anti-malarial efficacy was assessed using a standard four-day test (i e, at day 7) and blood parasitaemia was measured by fluorescence-activated cell sorting (FACS) analysis [38]. The limit of detection (per cent of *P. falciparum*) was 0.01%. The number of parasites ×10^6^ cells was recorded and data were analysed by non-linear fitting to a logistic equation of log_10_ (per cent parasitaemia at day 7 after infection) *versus* the dose level administered.

Per cent parasitaemia at day 7 after infection in treated *versus* control animals was analysed using a one factor ANOVA with Tukey’s post-test analysis. If there was a significant difference (*P* < .05) then the ED_50_ was calculated as the dose in mg/kg that reduced parasitaemia at day 7 after infection by 50% with respect to vehicle-treated mice. ED_90_ was calculated similarly. Analysis was performed using WinNonlin 5.2 and GraphPad Prism 5.0.

The pharmacokinetics of compounds after oral administration was determined concurrently in the same mice used for the therapeutic efficacy assay. Samples were taken at 0.25, 0.5, 1, 3, 6, 8, and 24 hours after the first dose. Compound levels were measured in 25 μL blood samples that were mixed with 25 μL of saponin (0.1% in water) and processed under standard liquid–liquid extraction conditions [39]. Pharmacokinetic parameters were calculated using WinNonlin 5.2 non-compartmental analysis. The data for the exposure of the drug in blood (area under the curve, AUC) after the first oral administration and parasitaemia at day 7 were fitted to a logistic function to predict the exposure necessary to inhibit parasitaemia at day 7 after infection in compound-treated mice by 90% with respect to vehicle-treated mice (AUC_ED90_).

## Results

### Screening

At SJCRH, screening of approximately 3,800 FDA-approved drugs and other bio-actives identified 24 compounds with EC_50_ values <1 μM (Table 2). Of these, 19 had known pharmacokinetic and/or safety profiles that were considered unsuitable for development as an oral anti-malarial drug. Of the other compounds, two are available only for topical/external use (dequalinium, demecarium); pravastatin cannot be used in pregnancy; and sulphamerazine is a sulphonamide – a class of molecule that has already yielded anti-malarial drugs, although *P. falciparum* has developed resistance to the compounds that are used clinically. Lestaurtinib (CEP-701) is a protein kinase inhibitor in development by Cephalon Inc for acute myelogenous leukaemia and myeloproliferative disorders. Clinical information on this compound was limited at the time of the study and protein kinase inhibitors have been suggested as an important target in malaria [9,40,41]. Thus, only lestaurtinib was progressed to the *P. falciparum* HuSCID mouse model. These results mirrored those previously reported by this group [42].

**Table 2:** Most active compounds tested by St Jude’s Children’s Research Hospital

**Compound**	**Class (therapeutic area)**	**EC**_**50 **_**3D7 (μM)**	**EC**_**50 **_**K1 (μM)**
Methylene blue	Nitric oxide/guanylate cyclase inhibitor (various)	<0.0003 (NA)	<0.0003 (NA)
Dactinomycin	Nucleoside reverse transcriptase inhibitor (oncology)	0.0009 (0, 0.13)	0.001 (0.0003, 0.006)
Sulfamerazine	Dihydrofolate synthetase inhibitor (anti-infective)	0.01 (0.01, 0.01)	0.01 (0.01, 0.01)
Methotrexate	Dihydrofolate reductase inhibitor (oncology)	0.01 (0.009, 0.01)	0.02 (0.01, 0.02)
Bortezomib	Proteasome inhibitor (oncology)	0.02 (0.01, 0.04)	0.08 (0.07, 0.09)
Thiothixene	Post-synaptic receptor agonist^a^ (anti-psychotic)	0.04 (0, 233.71)	0.02 (0.01, 0.05)
Dequalinium	Anti-septic	0.06 (0.002, 1.53)	0.06 (0.03, 0.12)
Doxorubicin	Topoisomerase II inhibitor, DNA intercalating agent (oncology)	0.21 (0.16, 0.27)	0.20 (0.14, 0.30)
Pentamidine	Inhibition of DNA, RNA, phospholipid and protein synthesis^b^ (anti-infective)	0.22 (0.18, 0.27)	0.05 (0.04, 0.06)
Bosutinib	Tyrosine kinase inhibitor (oncology)	0.22 (0.016, 3.11)	0.65 (0.36, 1.19)
Aminopterin	Dihydrofolate reductase inhibitor (oncology)	0.32 (0.30, 0.33)	1.25 (1.11, 1.41)
Midostaurin	Multi-kinase inhibitor (oncology)	0.35 (0.17, 0.71)	0.15 (0.13, 0.17)
Lestaurtinib	FMS-like tyrosine kinase 3 inhibitor (oncology)	0.49 (0.28, 0.84)	0.34 (0.29, 0.41)
Demecarium	Cholinesterase inhibitor (ophthalmology)	0.51 (0.45, 0.57)	0.30 (0.26, 0.36)
Cyproterone	Steroidal anti-androgen (oncology)	0.56 (0.54, 0.58)	0.89 (0, 1501.50)
Lapatinib	Tyrosine kinase inhibitor (oncology)	0.56 (0.39, 0.80)	>7.37 (NA)
Pimozide	Dopamine receptor blocker (anti-psychotic)	0.70 (0.44, 1.11)	>12.76 (NA)
Pravastatin	HMG-CoA reductase inhibitor (anti-cholesterol)	0.75 (0.51, 1.09)	0.12 (0.10, 0.15)
Dipyrone	NSAID^b^ (pain)	0.84 (0.71, 0.98)	0.50 (0.21, 1.16)
Mitomycin	Inhibition of DNA synthesis (oncology)	0.97 (0.81, 1.17)	0.51 (0.45, 0.57)
Propafenone	Sodium channel modulator (cardiology)	1.22 (0.58, 2.55)	0.33 (0.31, 0.34)
Cyclosporin A	Immune suppressant (oncology)	1.23 (1.06, 1.44)	0.87 (0.62, 1.23)
Vorinostat	Histone deacetylase inhibitor (oncology)	1.47 (1.17, 1.84)	0.84 (0.76, 0.93)
Sorafenib	Multi-kinase inhibitor (oncology)	2.71 (2.4, 3.1)	0.88 (0.7, 1.1)

NA, not available. EC_50_ values are shown as mean (95% CI). Compounds with an EC_50_ < 1 μM against either *P. falciparum* strain are shown. Relevant results from all compound sets tested at SJCRH are shown. Where compounds were duplicated across the consolidated test set, the results with the most potent *in vitro* activity are shown. Known anti-malarial drugs have been excluded.

^a^Agonist of dopaminergic-receptors, serotonergic-receptors, histaminergic-receptors, alpha1/alpha2-receptors, and muscarinic (cholinergic) M1/M2-receptors.

^b^Mechanism unknown.

SYBR®I fluorescence assay for activity against *P. falciparum* strains 3D7 and K1.

In the GSK discontinued drugs set, 6.4% (4/63) of compounds tested showed activity greater than 50% inhibition at a concentration of 2 μM in the [^3^H] hypoxanthine incorporation assay at 48 hours; IC_50_ values are shown in Table 3. Upon further evaluation, these four compounds were not progressed for the following reasons. Piritrexim is a dihydrofolate reductase inhibitor and lurtotecan a topoisomerase I inhibitor and neither molecule demonstrated a significant potential therapeutic window between inhibition of the parasite and inhibition of tumor-derived cell lines. GSK202405, a muscarinic receptor agonist, is delivered via oral inhaler and has limited oral availability. SB-435495 is a phospholipase A2-inhibitor of the pyrimidone class. Previous work with this series resulted in the clinical anti-malarial candidate GSK-932121, which was stopped in clinical development because of adverse events linked to human mitochondrial respiration. SB-435495 was, therefore, not continued because of a poor human/parasite selectivity window and, after EC_50_ determination, its *in vitro* activity was borderline (1.06 μM).

**Table 3:** **Most active compounds *in vitro *** **from the GlaxoSmithKline discontinued drugs compound set**

**Compound**	**Class (therapeutic area)**	**IC**_**50 **_**μM**
Piritrexim	Dihydrofolate reductase inhibitor (oncology)	0.011 ± 0.001
SB-435495	Phospholipase A2-inhibitor (anti-infective/anti-inflammatory)	1.126 ± 0.146
Lurtotecan	Topoisomerase I inhibitor (oncology)	0.191 ± 0.062
GSK202405	Muscarinic receptor agonist (asthma)	1.582 ± 0.206

Values are mean of three independent measures.

Study performed by GlaxoSmithKline.

IC_50_ determination against *P. falciparum* 3D7A strain using [^3^H] hypoxanthine incorporation assay.

For the Pfizer STLAR set, the initial HTS reported >50% activity against *P. falciparum* 3D7 and Dd2 at the 0.784 μM concentration for 1.7% (3/176) of compounds, with 13.6% (24/176) having activity ≥90% at a concentration of 7.84 μM. Further evaluation of 13 of the more active compounds, identified five with EC_50_ values <1 μM against either *P. falciparum* 3D7 or K1 (Table 4). UK-112,214 is a dual platelet activating factor receptor/histamine H1 (PAF-H1) receptor antagonist and was selected for *in vivo* studies in the *P. falciparum* mouse model. The other four compounds were not progressed for the following reasons (unpublished data provided by Pfizer Inc): CP-631992 is a neuropeptide Y5 receptor antagonist discontinued because of unfavourable animal toxicity findings; CE-245677 is a TIE2 tyrosine kinase inhibitor with reports of significant central nervous system adverse events at human plasma levels of ~1.5 μM; CJ-0231112 is a bradykinin B2 receptor antagonist and was rejected based on drug stability issues and the effect of food on absorption; and AG-024322, a CDK1/2/4/5 inhibitor, was known to have a narrow therapeutic window in mouse cancer models and demonstrated poor tolerability in Phase I studies.

**Table 4:** **Most active compounds *in vitro *** **from the Pfizer STLAR library**

**Compound**	**Class (therapeutic area)**	**EC**_**50 **_**3D7 (μM)**	**EC**_**50 **_**K1 (μM)**
UK-112,214	Dual platelet activating factor/histamine H1 receptor antagonist (allergic rhinitis)	0.55 (0.45, 0.65)	0.6 (NA)
CP-631992	Neuropeptide Y5 receptor antagonist (obesity)	0.7 (NA)	0.40 (0.2, 0.6)
CE-245677	TIE2 tyrosine kinase inhibitor (oncology)	1.1 (NA)	0.8 (NA)
CJ-0231112	Bradykinin B2 receptor antagonist (pain)	0.65 (0.36, 0.94)	0.4 (NA)
AG-024322	CDK1/2/4/5 inhibitor (oncology)	0.7 (0.11, 1.29)	0.4 (NA)

NA, not available. EC_50_ values are shown as mean (95% CI). Compounds with an EC_50_ < 1 μM against either *P. falciparum* strain are shown.

Study performed by Pfizer Inc.

SYBR® I fluorescence assay for activity against *P. falciparum* strains 3D7 and K1.

**Figure 2:**
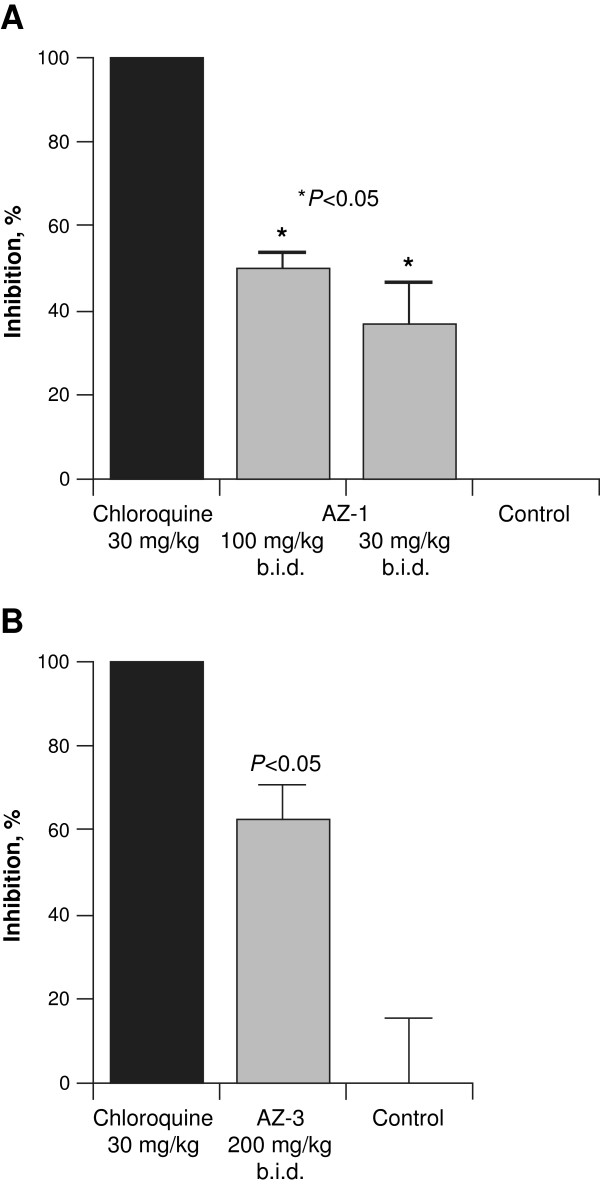
***In vivo *efficacy of (A) AZ-1 and (B) AZ-3 candidate drugs in *Plasmodium berghei*-infected mice.** Study performed by AstraZeneca. Male BALB/c (n = 3) mice infected with *P. berghei* intraperitoneally were treated simultaneously with different dose groups of compounds and controls for four days starting from day 0. The percentage growth inhibition on final day was plotted against different groups. *P* values are *versus* untreated controls.

For the AZ set, 6/100 compounds had an EC_50_ ≤ 1 μM (Table 5). All six compounds originated from oncology programmes, mainly targeting human kinases. Of these six compounds, AZ-4 targeting CDK2 and AZ-5 targeting aurora kinase were not progressed further because of toxicity concerns with these targets incompatible with an anti-malarial therapy, specifically the essential role of CDK2 in maintaining genomic stability in mammals and myelosuppression associated with aurora kinase inhibition [43,44]. AZ-6 was not progressed because of poor selectivity with respect to HepG2 cytotoxicity. AZ-1 and AZ-2 are very closely related structurally. AZ-1 targets the Trk1 potassium transporter and AZ-2 targets JAK2, though both compounds have potential cardiovascular issues via hERG regulation. AZ-3 emerged from an oncology programme targeting human farnesyl transferase. AZ-1 and AZ-3 were further investigated for efficacy against *P. berghei* with the aim that if the compounds showed efficacy, they could be considered as starting points for a lead optimization programme. Pharmacokinetic studies guided the selection of the 100 or 200 mg/kg BID dose used in the *in vivo* experiments. Oral aminobenzotriazole 100 mg/kg was administered to inactivate cytochrome P450 metabolism and increase drug bioavailability. However, both compounds were only marginally efficacious (50–60% inhibition) at high doses (100–200 mg/kg BID) (Figure 2). The lack of convincing efficacy even at high doses coupled with concerns regarding target selectivity and safety led to a halt in the further investigation of these compounds.

**Table 5:** **Most active compounds *in vitro *** **from the AstraZeneca discontinued drugs compound set**

**Compound**	**Target**	***Pf *** **EC**_**50 **_**(μM)**	**HepG2 EC**_**50 **_**(μM)**	**Status in original indication**
AZ-1	Trk1	0.6	10.4	Stopped after GLP toxicity
AZ-2	JAK2	0.1	2.0	Stopped after GLP toxicity
AZ-3	FAR	1.1	11.7	Stopped after Phase II
AZ-4	CDK2	1.2	11.3	Stopped after GLP toxicity
AZ-5	Aurora kinase 1	0.4	17.1	Stopped after GLP toxicity
AZ-6	CHK1	0.4	0.3	Stopped after GLP toxicity

GLP, good laboratory practice.

Study performed by AstraZeneca.

SYBR® I fluorescence assay for activity against *P. falciparum* strain NF54 and cytotoxicity assay against HepG2.

### *Plasmodium falciparum* huSCID mouse model

**Figure 3:**
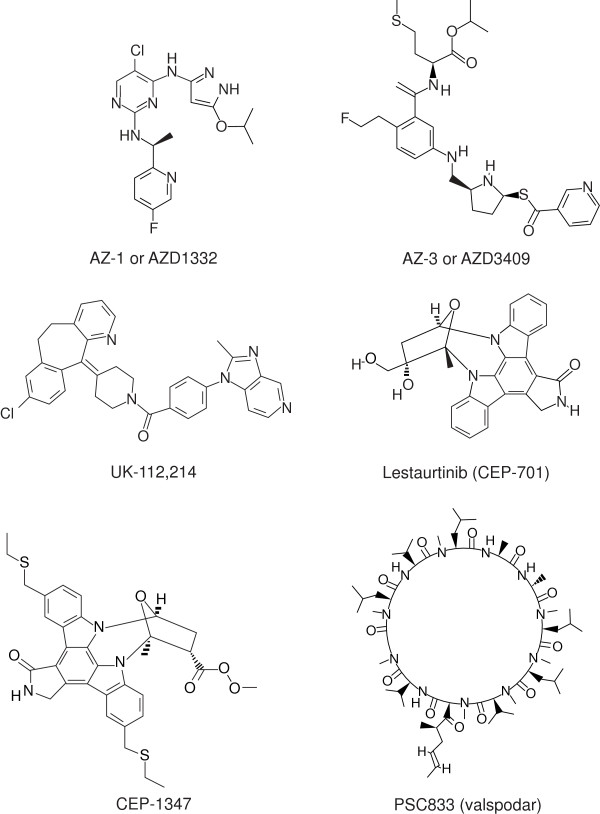
**Structure of four compounds tested in the *Plasmodium falciparum *** **huSCID mouse model and two compounds tested in *Plasmodium berghei*-infected mice.**

The *in vivo* efficacy of four compounds was determined against *P. falciparum* in the humanized mouse model (Table 6, Figure 3). Two of these (UK-112,214 and lestaurtinib) were identified in screening and two (CEP-1347 and PSC-833) were sourced additionally as a result of findings with related compounds during screening.

**Table 6:** **Therapeutic efficacy of test compounds against *Plasmodium falciparum *** **Pf3D7**^**0087/N9 **^**in a humanized mouse model**

**Compound**	**Target dose (mg/kg)/route**	**ED**_**50 **_**(mg/kg)**	**ED**_**90 **_**(mg/kg)**	**AUC**_**ED90 **_**(μg · h · mL**^**−1**^ **· day**^**−1**^**)**
UK-112,214	100, 300 po	80.1 (99.8, 55.1)	131.3 (112.3, 156.7)	111.5 (106.6, 121.1)
CEP-701	10, 30 sc	NC	NC	NC
CEP-1347	10, 30 sc	NC	NC	NC
PSC-833	50, 100, 200 po	–	>200	>17.33

ED_50_ and ED_90_ values are shown as the mean (95% CI). Data was calculated to three decimal places.

NC, not calculated as the percent reduction in parasitaemia was not significantly different from that of the control animals receiving vehicle only.

sc, subcutaneous; po, oral administration.

Study performed by GlaxoSmithKline.

**Figure 4:**
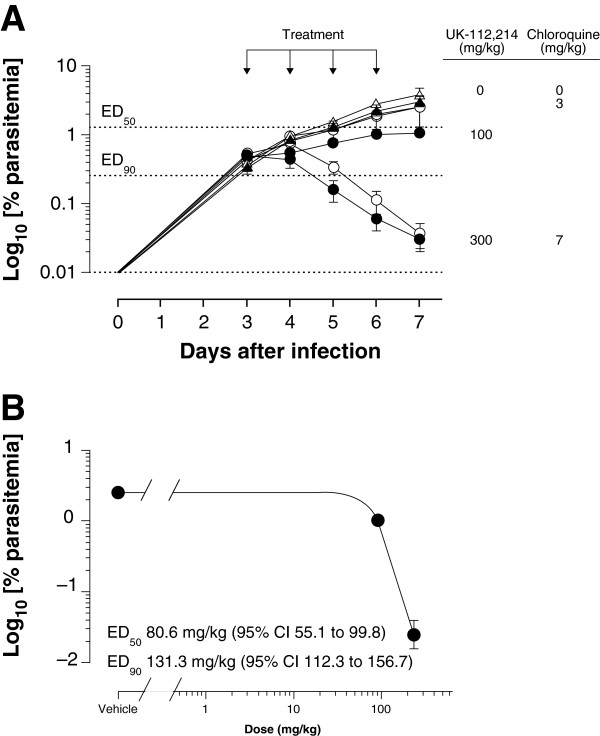
**Therapeutic efficacy of UK-112,214 against *Plasmodium falciparum *Pf3D7**^**0087/N9**^**. A)** Parasitaemia in peripheral blood of mice obtained from day 3 to day 7 after infection for UK-112,214 (closed circles) or chloroquine (open circles). Data are presented as mean of three mice ± SE for log_10_[% parasitaemia]. Data for vehicle-treated animals are denoted by triangles; **B)** Dose–response relationship for log_10_ [% parasitaemia] at day 7 after infection. Study performed by GlaxoSmithKline.

**Figure 5:**
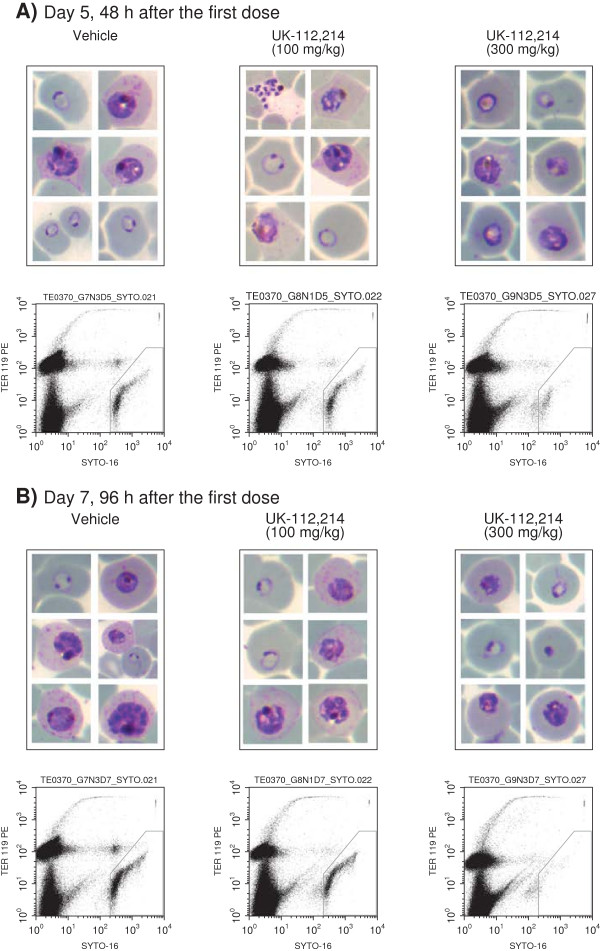
**The effect of UK-112,214 treatment on *Plasmodium falciparum *Pf3D70087/N9 *in vivo *at (A) Day 5 and (B) Day 7.** Photomicrographs of peripheral blood smears stained with Giemsa. Lower panels show flow cytometry dot plots from samples of peripheral blood stained with TER-119-Phycoerythrine (marker of murine erythrocytes) and SYTO-16 (nucleic acid dye). Dots inside the polygonal region represent *P. falciparum*-infected human erythrocytes. Study performed by GlaxoSmithKline.

The most active agent tested was UK-112,214, a water soluble PAF-H1 inhibitor identified in the Pfizer STLAR screen (Figure 4A). UK-112,214 had an ED_90_ of 131.3 mg/kg (95% CI 112.3, 156.7), oral exposure was good, and the pharmacokinetic profile appeared linear within the dosing range (Figure 4B, Table 7). Exposure data from UK-112,214-treated mice *versus* parasitaemia fitted a sigmoid function (coefficient of determination *R*^2^ = .996). The estimated AUC_ED90_ for UK-112,214 was 111.5 μg · h · mL^−1^ · day^−1^ (95% CI 106.6, 121.1) (Table 7). In this model, the ED_90_ or AUC_ED90_ mark the limit between *P. falciparum* net growth (at lower dose or exposure) or net clearance from peripheral blood (at higher doses or exposure). Therefore, in order to achieve net clearance of *P. falciparum* from peripheral blood of mice in two cycles of the parasite, a daily exposure higher than the AUC_ED90_ (111.5 μg · h · mL^−1^ · day^−1^) would be required. A qualitative analysis of the effect of treatment with 300 mg/kg UK-122,214 using microscopy and flow cytometry found parasites remaining in peripheral blood 48 hours after the start of treatment (Figure 5A). These showed cytoplasmic condensation, vacuolization of trophozoites and absence of mature schizonts. At 96 hours after the start of treatment some pycnotic parasites were also detected (Figure 5B). These results suggest that UK-112,214 does not induce fast killing of *P. falciparum* in peripheral blood.

**Table 7:** **Pharmacokinetics of test compounds in *Plasmodium falciparum*-infected humanized mice**

**Compound**	**Target dose (mg/kg)**	**C**_**max **_**(μg/mL)**	**t**_**max **_**(h)**	**AUC**_**(0–t) **_**(μg · h · mL)**	**DNAUC**_**(0–t) **_**(μg · h · mL**^**−1**^ **· day**^**−1**^**)**
UK-112,214	100	8.61 (0.4)	4.0 (1.7)	72.1 (2.7)	0.859 (0.32)
UK-112,214	300	17.6 (6.4)	3.3 (4.0)	231 (101)	1.03 (0.45)
CEP-701^a^	10	0.63 (0.079)	0.78 (0.38)	2.8 (0.46)	0.44 (0.072)
CEP-701^a^	30	2.8 (0.84)	4.0 (1.7)	23.8 (6.8)	1.6 (0.47)
CEP-1347^b^	10	0.83 (0.44)	4.8 (4.0)	8.1 (1.8)	0.98 (0.22)
CEP-1347^b^	30	0.73 (0.20)	6.0 (NA)	9.9 (2.1)	0.45 (0.092)
PSC-833^c^	50	1.39 (0.20)	3.30 (1.2)	12.90 (2.97)	0.36 (0.082)
PSC-833	100	1.01 (0.53)	5.33 (3.05)	12.26 (4.25)	0.13 (0.04)
PSC-833	200	0.91 (0.47)	2 (0)	13.05 (6.05)	0.065 (0.03)

NA, not available; DNAUC, dose-normalized value of AUC_(0–t)_.

Values are the mean (SD). Pharmacokinetics were estimated in whole blood after oral UK-112,214 and PSC-833 or subcutaneous CEP-701 and CEP-1347 administration to *P. falciparum*-infected humanized mice.

^a^Note that the experimental doses were 6.4 and 15.0 mg/kg (*versus* target doses of 10 and 30 mg/kg, respectively).

^b^Note that the experimental doses were 8.3 and 22.0 mg/kg (*versus* target doses of 10 and 30 mg/kg, respectively).

^c^Note that the experimental dose was 36 mg/kg (*versus* target dose of 50 mg/kg).

Study performed by GlaxoSmithKline.

**Figure 6:**
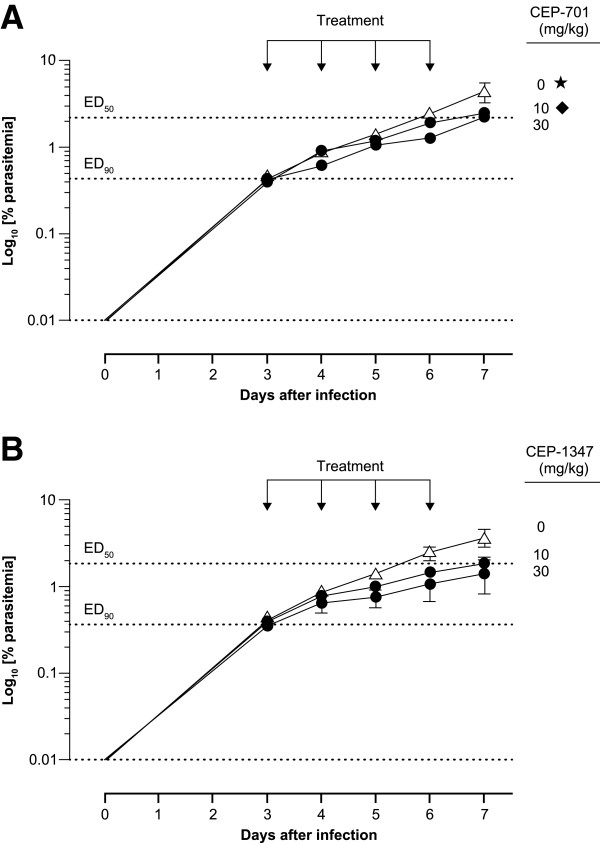
**Therapeutic efficacy of A) lestaurtinib (CEP-701) and B) CEP-1347 against *Plasmodium falciparum *Pf3D7**^**0087/N9**^**.** Parasitaemia in peripheral blood of mice obtained from day 3 to day 7 after infection, data for vehicle-treated animals are denoted by triangles. Data are presented as mean of three mice ± SE for log_10_ [% parasitaemia] except in plot A, where groups labelled with symbols had two mice (*) or one mouse (♦) at the end of the experiment. Study performed by GlaxoSmithKline.

Lestaurtinib (CEP-701) is a protein kinase inhibitor thought to target fibroblast growth factor receptor 1 (FGFR1), fms-like tyrosine kinase 3 (FLT3), tyrosine kinase A (TrkA) and janus kinase 2 (Jak2). A related compound (CEP-1347) was also provided by Cephalon Inc for testing in the model. These compounds were tested up to the maximum tolerated dose. Although there was a trend for reduced parasitaemia in mice treated with these compounds, the reduction did not reach statistical significance and ED_90_ or AUC_ED90_ could not be estimated (Figure 6). For CEP-1347 in the *P. falciparum-*infected mice, the pharmacokinetics after subcutaneous administration in the studied dose range did not appear to be linear, with similar values of C_max_ and AUC _(0–t)_ after the administration of the two selected doses (Table 7). The experimental doses of lestaurtinib were lower than the target ones, but again, non-linear pharmacokinetic behaviour was observed (Table 7). Note that preclinical studies in mouse cancer models had shown efficacy at exposures similar to those that were achieved in the current study [45].

An additional compound, PSC-833 (valspodar), was tested. This is a non-immunosuppressive cyclosporin derivative developed primarily as a p-glycoprotein inhibitor. As cyclosporin had been active during *in vitro* screening against *P. falciparum* but cannot be considered because of its immunosuppressive properties, valspodar was considered a potential substitute for addressing the cyclosporin target. This compound was sourced from Novartis AG, and although it had completed Phase III studies as an oncology drug, it had been discontinued for lack of efficacy. Valspodar did not significantly inhibit *P. falciparum* parasitaemia *in vivo* (ED_90_ > 200 mg/kg) (Table 6). The oral pharmacokinetics in the dose range studied was non-linear, with similar values of AUC_(0–t)_ for both dose levels (100 and 200 mg/kg) (Table 7).

In programmes that are currently being conducted in collaboration with or supported by MMV, a significant *in vivo* potency in the humanized mouse model is considered to be lower than 20 mg/kg. Therefore, none of the drugs tested met the criteria for further development.

## Discussion

Although a large number of approved, investigational and discontinued drugs were evaluated in this project, none of the compounds identified with antiplasmodial activity met the candidate selection criteria warranting further development. From the approximately 3,800 compounds that were tested by SJCRH, there were 24 with EC_50_ values <1 μM against *P. falciparum* – a hit rate of about 0.6%, which is similar to that obtained when testing sets of random pharmaceutical diversity. Within the unregistered compound sets of GSK, Pfizer and AZ, 15 of the 338 compounds tested showed significant *in vitro* activity – a hit rate of 4.4%. This higher hit rate in the unregistered compound sets probably reflects the greater diversity of bio-activity the SJCRH compound set. The unregistered compounds reflect the focus of recent pharmaceutical development in the companies concerned in anti-proliferative, anti-infective and anti-inflammatory disease, areas likely to have biological overlap with processes in the malaria parasite.

Encouragingly, it is clear that a number of different targets in the malaria parasite can be addressed by existing drugs. For example, several protein kinase inhibitors showed *in vitro* activity against *P. falciparum* in this study (bosutinib, midostaurin, lestaurtinib, lapatinib, sorafenib, and CE-245677). These compounds were of particular interest as they are essential throughout all stages of the *Plasmodium* spp. lifecycle [40,41]. Many protein kinase inhibitors have been registered or investigated, primarily for the treatment of cancer, although these drugs have known toxicities that have discouraged their use in malaria. Antiretroviral protease inhibitors were also of interest and tested in this study, though they had relatively poor *in vitro* activity. Previous data showed moderate *in vitro* activity of saquinavir, nevirapine, ritonavir, nelfinavir, amprenavir, and indinavir at clinically relevant concentrations [46]. However, a recent clinical study in HIV-infected women from malaria-endemic regions of sub-Saharan Africa showed no effect of antiretroviral treatment on the incidence of malaria [47].

Among the licensed products that were active *in vitro*, none of the compounds were progressed to the *in vivo* model, mainly because of their unfavourable pharmacokinetic and/or safety profile for use as an oral anti-malarial. However, the scope of this study did not include speculation about the clinical safety and pharmacokinetics that *might* be discovered *should* clinical studies in malaria be conducted. In fact, a number of these compounds have been investigated further in malaria. Methotrexate has good activity against *P. falciparum* and *Plasmodium vivax in vitro*, although poor activity *in vivo* against murine malaria species [48-50]. The assumed toxicity of methotrexate and other anticancer drugs when used in short-course, low-dose therapy has been questioned [51]. However, a recent clinical study of methotrexate in healthy volunteers failed to achieve sufficient drug exposures for effective malaria therapy [52]. Methylene blue has also been investigated clinically for malaria, although it is slow acting and there are potential haemolytic effects of this compound in glucose-6-phosphate dehydrogenase-deficient individuals [53-56]. Bortezomib has confirmed *in vitro* activity against *P. falciparum*[57], although clinically its effect as an immunosuppressant probably precludes its use in malaria. Similarly, although cyclosporin A has shown good efficacy in a murine mouse model [58], its immunosuppressive effect prevents its repositioning as an anti-malarial.

**Figure 7:**
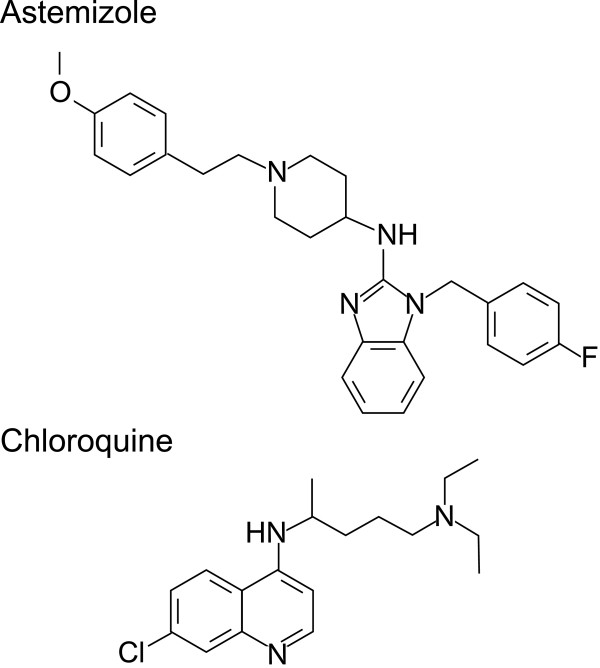
Chemical structures of astemizole and the 4-aminoquinoline ring (chloroquine).

Of the non-marketed products, four were selected from the test sets for *in vivo* evaluation (AZ-1, AZ-3, UK-112,214, and lestaurtinib) and two further drugs were sourced directly from their respective patent owners, CEP-1347 from Cephalon Inc and PSC833 (valspodar) from Novartis Inc. Of these six compounds, only UK-112,214 showed significant activity *in vivo*. UK-112,214 is a water soluble PAF-H1 inhibitor targeted for use in allergic inflammatory conditions, such as allergic rhinitis. This is perhaps an unexpected target as clinical studies of the role of PAF in the most severe form of malaria, cerebral malaria, have been inconclusive [59]. However, astemizole, identified as a promising compound for repositioning in a previously reported study, is also a PAF-H1 inhibitor [22]. Of interest is that both UK-112,214 and astemizole have chemical structures related to known anti-malarial drugs of the 4-aminoquinoline class (Figures 3 and 7) and do not, therefore, represent a new class of anti-malarial agent. Astemizole was withdrawn because of cardiovascular adverse events, specifically prolongation of the QT interval caused by potent inhibition of hERG potassium channels [60]. The relative potential for cardiovascular adverse events with UK-112,214 is so far unreported, but an independently run hERG assay suggests it may too have a cardiac liability (hERG = 2.8 μM). The rate of *P. falciparum* parasite killing with UK-112,214 was slow, though it could potentially have utility as a combination therapy for the treatment of asexual *P. falciparum* should sufficient human exposure levels be achieved. Unfortunately, there are no human pharmacokinetic data on this compound in the public domain, but single-dose pharmacokinetic data provided by Pfizer indicate that UK-112,214 at doses from 10 mg to 480 mg resulted in C_max_ values from 14 to 4145 ng/ml.

Safety is the greatest impediment to the repositioning of existing drugs to treat malaria. Anti-malarial drugs are taken in possibly many millions of doses every year. Most importantly, an anti-malarial must be safe in children and pregnant women as these groups are most severely affected by the disease. Supply to the patient is often unregulated, self-medication is common and medical resources may be limited. Thus, patients may not be monitored for adverse events or be able to access medical care should these occur.

To achieve the required therapeutic window for an anti-malarial drug, it should have good oral bio-availability, potent activity against the parasite and a high specificity for perturbing parasite metabolic and biochemical processes *versus* those of the host, ie, few and mild adverse events. These requirements are challenging, particularly for drugs that have been developed to affect human disease processes. In general, unless a drug demonstrates efficacy in malaria at a lower dose than in the ‘parent’ indication, the required therapeutic window cannot be achieved. Thus, repositioning of clinical compounds would seem most appropriate when the new use has a higher tolerance of potential safety signals, such as from malaria to cancer chemotherapy rather than *vice versa*.

In fact, anti-malarial drugs have been successfully repositioned into other therapeutic areas. Classically, hydroxyl chloroquine has been used to treat inflammatory conditions such as systemic lupus erythematosus, lupus nephritis and rheumatoid arthritis [61], and may also have utility in other auto-immune diseases [62]. More recently, investigations have been initiated into the use of anti-malarial drugs in cancer, for example, for the sensitization of tumours to enhance the response to conventional treatments [63,64]. Schistosomiasis is another indication that is being examined [65]. In particular, artemisinins appear to have many potential uses in diverse indications [66].

## Conclusions

In recent years, repositioning of existing drug therapy has been suggested as a fast track to developing new anti-malarial medicines [51,67,68]. Studies such as this are necessary in the continuing efforts to explore all potential routes in the search for new effective medicines against this devastating disease. However, the drugs tested in this study did not approach the efficacy requirements for progression or had known safety issues preventing their use in malaria. Thus, it is becoming evident that the development of new drugs for the treatment of uncomplicated *P. falciparum* infection will probably require the design of molecules specifically targeted at the parasite and pharmacokinetically optimized to provide a sufficient margin of safety.

## Competing interests

TP, JM and TW are employees of Medicines for Malaria Venture. JL is a former employee of Medicines for Malaria Venture. FJG-B, IA-B and SF-B are employees of GlaxoSmithKline Plc. SB is an employee of AstraZeneca. PV, BB and NR are former employees of AstraZeneca. TP is a former employee of Pfizer Inc. The remaining authors declare no competing interests.

## Authors’ contributions

TW generated the study concept. TW, JL and JM planned and coordinated the study. FJG-B, IA-B, JC, MC, SF-B, TP, PV, BB, NR, SB, SD, VA, and RKG were involved in data acquisition and data analysis. RKG provided expert advice on the *in vitro* screening strategy. All authors contributed to the paper and read and approved the final version submitted.

## Supplementary Material

Additional file 1 Expanded methods for in vitro assays. Click here for file
