# Supplementary material for: Repositioning: the fast track to new anti-malarial medicines?
Source: Malar J. 2014 Apr 14;13:143. doi: 10.1186/1475-2875-13-143 (PMC4021201; doi:10.1186/1475-2875-13-143)
Supplement: Additional file 1 — Expanded methods for in vitro assays. [file 1475-2875-13-143-S1.doc]

## Lotharius, et al. Additional file 1: Expanded methods for in vitro assays

**St Jude’s Children’s Research Hospital (SJCRH).** This group used the SYBR® I dye DNA staining assay, which measures proliferation of *P. falciparum* in human erythrocytes [1]. *Plasmodium falciparum* strains 3D7 (chloroquine-sensitive) and K1 (chloroquine-resistant) (American Type Culture Collection [ATCC], Manassas, VA) were maintained in group O+ erythrocytes (Key Biologics, LLC, Memphis, TN) using established methods [2]. Serial dilutions of the test compound in DMSO were prepared to provide 10 concentrations for each compound. 60 nL of test compound dilution were incubated with 20 µL of a synchronized culture suspension (1% rings, 4% hematocrit) and 20 µL of RPMI 1640 with 5 µg/mL gentamycin in a 384-well assay plate for 72 h. After addition of 10 µL mixed DNA dye solution in PBS per test well (10× SYBR® I, [Invitrogen, Carlsbad, CA]), 0.5% [vol/vol] Triton X-100, 0.5 mg/mL saponin), plates were shaken for 1 min at 750 rpm and incubated in the dark for 90 min. Fluorescence was measured with an EnVision®spectrophotometer (PerkinElmer, Waltham, MA) [3]. Tests were run in triplicate in two independent runs to generate 10-point dose–response curves to determine the half maximal effective concentration (EC_50_) against the 3D7 and K1 *P. falciparum* strains for each drug. EC_50_ values were calculated with the robust investigation of screening experiments (RISE) algorithm with a four-parameter logistic equation. EC_50_ values of <1μM were considered significant.

**GlaxoSmithKline, Tres Cantos.** A whole-cell [^3^H]hypoxanthine radioisotope incorporation assay was performed to determine percent parasite inhibition at 48 h and 96 h [4, 5]. *Plasmodium falciparum* 3D7A strain (Malaria Research and Reference Reagent Resource Center MR4; http://www.mr4.org), was maintained in human erythrocytes (Transfusions Blood Bank, Madrid) with Albumax II medium, as previously described [5]. Parasite growth inhibition assays and EC_50_ determination were carried out following standard methods [5]. Briefly, cultures with 0.5% parasitemia and 2.5% hematocrit were incubated with the 2 μM of test drug for 48 h or 96 h. [^3^H]hypoxanthine (0.8 μCi/mL) in 10 μL of RPMI was added 24 h before parasites were harvested (Cell Harvester 96™, TOMTEC, Hamden, CT). Incorporated radioactivity was quantified with a Wallac MicroBeta® TriLux (Model 1450 LS, PerkinElmer). Three independent experiments were conducted for each time duration and test compound. Inactive and active controls were also included. Parasite inhibition of ≥50% at 48 h relative to non-treated parasitized controls was considered significant.

**Discovery Biology, Eskitis Institute.** The initial high-throughput screen of the Pfizer STLAR set was performed by Discovery Biology, Griffith University, Nathan, Australia using a DNA-binding assay using 4′,6-diamidino-2-phenylindole (DAPI). This method has been recently described in detail elsewhere [6]. Briefly, *P. falciparum* 3D7 and the Dd2 clone, that has a high propensity to acquire drug resistance were maintained in O+ human erythrocytes (Australian Red Cross Blood Service, agreement no: 09-05QLD-06) using standard methods with some adaptations [2, 6]. 384-well imaging plates were prepared with a final assay well volume of 50 μL, containing 2% or 3% parasitemia and 0.3% hematocrit plus 0.4% DMSO only (minimum inhibition control), 0.4% DMSO with 2 μM artemisinin (maximum inhibition control) or test compound at the concentrations of 7.84 μM (duplicated runs), 0.784 μM or 0.0784 μM. Plates were incubated for 72 h at 37°C, 5% CO_2_ before restoring to room temperature and undergoing the staining procedure with DAPI (FluoroPure™ grade, Invitrogen, Carlsbad, CA) [6]. Plates were imaged with an Opera confocal microplate imaging reader (PerkinElmer) (405 nm excitation/ 420–490 nm emission) and a Victor^2^™ microplate reader (PerkinElmer) (355 nm excitation/ 460 nm emission). Inhibition values of treated wells (T) were calculated relative to the minimum and maximum inhibition controls [6]. Inhibition of ≥50% at a concentration of 0.784 μM was considered significant.

**Pfizer.** Following the HTS of the Pfizer STLAR compound set as described above, EC_50_ values were determined for a sub-set of active compounds by Pfizer using a SYBR® I dye DNA staining assay, similar to that described for SJCRH above. Cultures of *P. falciparum* 3D7 and K1 (both from David Baker, LSHTM) were maintained in O+ human erythrocytes (Research Red Cells and Blood Transfusion Service). 384-well assay plates contained 250 nL of compound at a serial dilution of 1:3 in 100% DMSO, starting from 200 µM (in the plate i.e. 20 µM final) and minimum and maximum controls. After addition of 50 μL of infected erythrocytes per well, plates were incubated at 37°C for three days in 5% CO_2_, 5% O_2_ and 90% N_2_. Each well then received 40 μL of SYBR® I (1:5000 dilution in lysis buffer) and was incubated at 37°C for at least 2 h. Plates were read using a Victor^2^™ microplate reader (485 nm excitation/ 528 nm emission). Percent anti-malarial activity was calculated relative to the minimum and maximum controls for each of the 11 drug concentrations and EC_50_ values determined from the resulting data plot.

**AstraZeneca.** This group also used a SYBR® I IC_50_ determination assay. Compounds were serially diluted in 384 well plates (C3570, Corning Life Sciences). 1 µL of compound was dispensed in a 10 point concentration response format using a Biomek Fx liquid dispenser. To this, 50 µL of *P. falciparum* NF54 (MRA-1000, MR4, ATCC, Manassas, Virginia) ring stage culture at 2% parasitemia and 0.3 % hematocrit was added using a multidrop dispenser. Plates were incubated at 37 ºC for 72 h in 5% CO_2_, 3% O_2_ and 92% N_2_ (Nuaire). After incubation, the % parasitemia in one of the untreated culture control wells was determined by light microscopy. In general, the % parasitemia at the end of the assay ranged from 8–12 % and the cultures had progressed to the trophozoite stage. 10 µL of SYBR® I lysis buffer (20 mM Tris-HCl pH 7.5, 10 mM EDTA, 0.16% saponin, 1.6% Triton X-100, 20× SYBR® I) was then added to all wells using the multidrop dispenser. Plates were incubated at room temperature for 24 h and then centrifuged at 4000 rpm for 30 min. 45 µL of the supernatant was aspirated and 50 µL of phosphate buffer saline was added and mixed using an Apricot personal pipette system (Apricot Designs). Green fluorescence (485 nm excitation/ 530 nm emission) was measured using the Safire 2 fluorimeter (Tecan Instruments). The % inhibition with respect to the control was plotted against the logarithm of the drug concentration. The curve was fitted by non-linear regression using the sigmoidal dose–response (variable slope) formula to yield the concentration response curves. The concentration at which 50% inhibition was observed was taken as the IC_50_ value of the compound.

The human hepatoma Hep G2 cell line was used for the cytotoxicity assay. 1 µL of compound was dispensed in a 10 point concentration response format using a Biomek Fx liquid dispenser into white sterile 384-well opaque bottom plates (C3570, Corning Life Sciences). To this, 50 µL of Hep G2 cells at 3000 cells/well was added using a Multidrop dispenser. The plates were then incubated at 37ºC for 72 h in a 5% CO_2_ incubator. Resazurin at a final concentration of 10 µM was added to all wells and the plates were incubated at room temperature for 4 h. Fluorescence (excitation 535 nm; emission 590 nm) was measured in a Safire 2 fluorimeter. The % inhibition with respect to the control was plotted against the logarithm of the drug concentration. The curve was fitted by nonlinear regression using the sigmoidal dose-response (variable slope) formula to yield the concentration response curves. The concentration at which 50% inhibition was observed was taken as the IC_50_ value of the compound.

**References**

1. Bennett TN, Paguio M, Gligorijevic B, Seudieu C, Kosar AD, Davidson E, Roepe PD: **Novel, rapid, and inexpensive cell-based quantification of antimalarial drug efficacy**. *Antimicrob Agents Chemother* 2004, **48**:1807-1810.

2. Trager W, Jensen JB: **Human malaria parasites in continuous culture**. *Science* 1976, **193**:673-675.

3. Smilkstein M, Sriwilaijaroen N, Kelly JX, Wilairat P, Riscoe M: **Simple and inexpensive fluorescence-based technique for high-throughput antimalarial drug screening**. *Antimicrob Agents Chemother* 2004, **48**:1803-1806.

4. Chulay JD, Haynes JD, Diggs CL: ***Plasmodium falciparum*: assessment of *in vitro* growth by [3H]hypoxanthine incorporation**. *Exp Parasitol* 1983, **55**:138-146.

5. Desjardins RE, Canfield CJ, Haynes JD, Chulay JD: **Quantitative assessment of antimalarial activity *in vitro* by a semiautomated microdilution technique**. *Antimicrob Agents Chemother* 1979, **16**:710-718.

6. Duffy S, Avery VM: **Development and optimization of a novel 384-well anti-malarial imaging assay validated for high-throughput screening**. *Am J Trop Med Hyg* 2012, **86**:84-92.
